# The electronic frailty index and outcomes in patients with myocardial infarction

**DOI:** 10.1093/ageing/afae150

**Published:** 2024-07-16

**Authors:** Matthew T H Lowry, Dorien M Kimenai, Dimitrios Doudesis, Konstantin Georgiev, Michael McDermott, Anda Bularga, Caelan Taggart, Ryan Wereski, Amy V Ferry, Stacey D Stewart, Christopher Tuck, David E Newby, Nicholas L Mills, Atul Anand

**Affiliations:** BHF Centre for Cardiovascular Science, University of Edinburgh, Edinburgh, UK; BHF Centre for Cardiovascular Science, University of Edinburgh, Edinburgh, UK; BHF Centre for Cardiovascular Science, University of Edinburgh, Edinburgh, UK; Usher Institute, University of Edinburgh, Edinburgh, UK; BHF Centre for Cardiovascular Science, University of Edinburgh, Edinburgh, UK; BHF Centre for Cardiovascular Science, University of Edinburgh, Edinburgh, UK; BHF Centre for Cardiovascular Science, University of Edinburgh, Edinburgh, UK; BHF Centre for Cardiovascular Science, University of Edinburgh, Edinburgh, UK; BHF Centre for Cardiovascular Science, University of Edinburgh, Edinburgh, UK; BHF Centre for Cardiovascular Science, University of Edinburgh, Edinburgh, UK; BHF Centre for Cardiovascular Science, University of Edinburgh, Edinburgh, UK; BHF Centre for Cardiovascular Science, University of Edinburgh, Edinburgh, UK; BHF Centre for Cardiovascular Science, University of Edinburgh, Edinburgh, UK; BHF Centre for Cardiovascular Science, University of Edinburgh, Edinburgh, UK; Usher Institute, University of Edinburgh, Edinburgh, UK; BHF Centre for Cardiovascular Science, University of Edinburgh, Edinburgh, UK

**Keywords:** frailty, myocardial infarction, electronic frailty index, routine data, older people

## Abstract

**Background:**

Frailty is increasingly present in patients with acute myocardial infarction. The electronic Frailty Index (eFI) is a validated method of identifying vulnerable older patients in the community from routine primary care data. Our aim was to assess the relationship between the eFI and outcomes in older patients hospitalised with acute myocardial infarction.

**Study design and setting:**

Retrospective cohort study using the DataLoch Heart Disease Registry comprising consecutive patients aged 65 years or over hospitalised with a myocardial infarction between October 2013 and March 2021.

**Methods:**

Patients were classified as fit, mild, moderate, or severely frail based on their eFI score. Cox-regression analysis was used to determine the association between frailty category and all-cause mortality.

**Results:**

In 4670 patients (median age 77 years [71–84], 43% female), 1865 (40%) were classified as fit, with 1699 (36%), 798 (17%) and 308 (7%) classified as mild, moderate and severely frail, respectively. In total, 1142 patients died within 12 months of which 248 (13%) and 147 (48%) were classified as fit and severely frail, respectively. After adjustment, any degree of frailty was associated with an increased risk of all-cause death with the risk greatest in the severely frail (reference = fit, adjusted hazard ratio 2.87 [95% confidence intervals 2.24 to 3.66]).

**Conclusion:**

The eFI identified patients at high risk of death following myocardial infarction. Automatic calculation within administrative data is feasible and could provide a low-cost method of identifying vulnerable older patients on hospital presentation.

## Key Points

- Frailty is common in acute myocardial infarction.
- The electronic frailty index can identify patients at high risk of adverse health outcomes following myocardial infarction.
- The electronic frailty index could be used to guide targeted frailty interventions in myocardial infarction.

## Introduction

Developing effective methods to manage an ageing and increasingly frail society represents one of the greatest challenges facing healthcare systems. This is particularly relevant for cardiology services: cardiovascular disease is the most prevalent condition in older adults with the majority of patients who suffer a myocardial infarction over the age of 70 years [1–3].

Frailty, a state of increased vulnerability, is common amongst patients with cardiovascular disease and is a strong independent predictor of poor clinical outcomes in numerous cardiovascular conditions, including myocardial infarction [4–7]. Guideline-recommended management recommends the use of individualised risk stratification with consideration of factors beyond a patient’s chronological age, including frailty [7, 8]. Despite this, frailty is rarely systematically or objectively measured, nor routinely used to identify high risk patients. This may in part be due to the challenges of frailty assessment in acute illness, where a patient’s ability to perform physical or mental tasks may be impaired [9]. Comprehensive assessment can be time-consuming, requiring additional resources, and subjective clinician assessment may overestimate frailty in cardiovascular patients, with agreement shown to vary based on clinician experience [10, 11].

The electronic Frailty Index (eFI) uses primary care data to identify and classify frailty. The eFI has been validated in large community-based populations aged 65 years old and over as a predictor of unplanned hospital admission and all-cause mortality at 1, 3 and 5 years, with good correlation with in-person frailty assessment [12–15]. However, the performance of the eFI in those hospitalised with myocardial infarction, and its ability to predict individual patient outcomes in an acute setting, is unclear.

Our aim was to evaluate the association between frailty, identified and stratified using the eFI, and the management and outcomes of patients admitted to hospital with myocardial infarction.

## Methods

### Study design and population

We conducted a retrospective observational cohort study using routinely collected electronic healthcare data collated in the DataLoch Heart Disease Registry. This registry consists of patients with cardiovascular disease in the South-East of Scotland [16]. Consecutive patients admitted to hospital with a diagnosis of myocardial infarction between October 1st 2013 and March 1st 2021 were included. Myocardial infarction was defined using International Classification of Disease (ICD-10) code I21 or I22 recorded in position one or two of the discharge coding [17]. In the case of multiple hospital admissions, the index event was defined as the earliest recorded episode. Patients under the age of 65 years on the date of index presentation were excluded.

The study was performed with approval of the local Research Ethics Committee and Caldicott Guardian in accordance with the Declaration of Helsinki.

### Electronic frailty index

Primary care Read codes (a standardised coding system for recording patient characteristics, symptoms, signs, diseases, disabilities, laboratory test results, and information about social circumstances) were used to calculate the eFI 24 h prior to the index hospital admission [12]. Read codes were used to identify the presence or absence of 36 deficits grouped across four domains: eighteen disease states, nine symptoms or signs, eight markers of disability, one abnormal laboratory value (**Appendix**). The number of deficits present per patient was divided by 36 to produce a score from 0 to 1. A patient was deemed frail if the score was ≥0.12 with the degree of frailty further divided in to mild (0.12–0.24), moderate (0.25–0.36) and severe (>0.36) [12].

### Study outcomes

The primary outcome was all-cause mortality at 12 months from the index presentation. Secondary outcomes included: in-patient all-cause mortality; unplanned hospital admission due to non-fatal myocardial infarction or urgent coronary revascularisation, heart failure, stroke, or major bleeding within 12 months of discharge; cardiovascular death at 12 months; and all-cause mortality at 3-years.

### Data sources

National data registering deaths (National Records of Scotland), medical prescriptions (Prescribing Information System) and inpatient activity (Scottish Morbidity Record) were used to identify outcomes. The Charlson Comorbidity Index was calculated using ICD-10 codes [18]. Provision of pharmacological therapies were determined from community prescription records at 30-days post-discharge. To reduce survival bias, all reporting of medical prescriptions was restricted to patients alive at this time point. Hospital admission with recurrent myocardial infarction, heart failure, stroke or major bleeding were identified using HDRUK phenotypes [19]. Major bleeding was defined as the occurrence of any bleed meeting the Bleeding Academic Research Consortium (BARC) Type 3 or Type 5 criteria [20]. Cardiovascular death was defined where any of the following ICD-10 codes were listed as the primary cause of death: I10-I15, I20-I25, I44-I51, I61, I62.0, I62.9, I63.0-I63.5, I63.8, I63.9, I64-I67 and I70-I73.

### Statistical analysis

Baseline characteristics were stratified according to eFI categories. Continuous variables were described using mean and standard deviation (SD) or median and 25th–75th centile where skewed. Categorical variables were presented as absolute numbers and percentages (%). Comparisons were performed using the Wilcoxon rank sum test, Kruskal–Wallis rank sum test and Pearson’s Chi-squared test where appropriate. Any categorical variables with a frequency of less than 5 are reported as ‘<5’ due to data protection requirements.

Univariable and multivariable Cox proportional hazard models were used to assess the relationship between eFI categories and all-cause death as an inpatient, at 12 months and at 3 years, and cardiovascular death at 12 months. For the outcome of cardiovascular death, the competing risk of non-cardiovascular death was accounted for using a Fine-Gray sub distribution hazard model [21]. Univariable and multivariable logistic regression was used to assess the relationship between frailty category, inpatient mortality, and unplanned hospital admission. We estimated all models with and without adjustment for age, sex, myocardial infarction classification (non-ST elevation vs ST-elevation myocardial infarction) and maximal cardiac troponin value recorded during the index admission. [22] Cardiac troponin was measured using the ARCHITECT*_STAT_* high-sensitivity troponin I assay and maximal troponin values were log transformed (log base 10).

Discrimination of the eFI score for the primary outcome was determined by calculating the area under the receiver operating characteristic curve (AUC) with calibration assessed visually using a calibration plot.

In subgroup analysis, we assessed the associated hazard of eFI categories by age (<75 vs ≥75 years), sex (male vs female) and the presence or absence of ST-segment elevation including testing for interaction.

All analysis was performed using remote access to deidentified data within a Secure Data Environment (DataLoch, Edinburgh, United Kingdom) and conducted using R (version 4.2.0; The R Foundation for Statistical Computing).

## Results

### Study population

A total of 4670 of the 8038 identified patients were eligible for inclusion after those younger than 65 years (*n* = 3368) were excluded. The median follow-up time was 3.0 (IQR, 1.1 to 5.3) years.

Of the 4670 patients (median age 77 [71–84] years, 43% female, 83% white), a total of 1865 (40%) were classified as fit with 1699 (36%), 798 (17%) and 308 (7%) classified as mild, moderate and severely frail respectively. Frail patients were older and more likely to be female (Table 1). Compared with fit patients, patients with severe frailty had a significantly greater prevalence of cardiovascular and non-cardiovascular comorbidities including previous myocardial infarction (41% versus 11%), heart failure (46% versus 4%), chronic kidney disease (45% versus 15%), dementia (19% versus 2%) and a Charlson Comorbidity Index of 2 or more (46% versus 3%, *P* < .01 for all).

**Table 1:** Baseline characteristics and patient management

	Overall	electronic Frailty Index Category				
		Fit	Mild	Moderate	Severe	*P*-value
*N*	4670	1865	1699	798	308	
Patient demographics						
Age (years)	77 [71–84]	72 [68–78]	79 [72–85]	81 [75–86]	82 [78–88]	<.001
Female	2014 (43%)	645 (35%)	784 (46%)	406 (51%)	179 (58%)	<.001
White	3865 (83%)	1419 (76%)	1449 (85%)	707 (89%)	290 (94%)	<.001
Deprivation (SIMD 1^st^ quintile)	648 (14%)	204 (11%)	235 (14%)	148 (19%)	61 (20%)	<.001
Chest pain as presenting symptom	3680 (82%)	1567 (87%)	1326 (81%)	568 (74%)	219 (73%)	<.001
Past medical history						
Ischaemic heart disease	1989 (43%)	418 (22%)	805 (47%)	522 (65%)	244 (79%)	<.001
Myocardial infarction	1061 (23%)	213 (11%)	434 (26%)	287 (36%)	127 (41%)	<.001
Heart failure	772 (17%)	68 (3.6%)	274 (16%)	288 (36%)	142 (46%)	<.001
Cerebrovascular disease	838 (18%)	98 (5.3%)	323 (19%)	277 (35%)	140 (45%)	<.001
Peripheral vascular disease	558 (12%)	73 (3.9%)	227 (13%)	173 (22%)	85 (28%)	<.001
Diabetes mellitus	1139 (24%)	206 (11%)	446 (26%)	325 (41%)	162 (53%)	<.001
Chronic kidney disease	1249 (27%)	286 (15%)	494 (29%)	331 (41%)	138 (45%)	<.001
Hypertension	2964 (63%)	930 (50%)	1029 (61%)	497 (62%)	187 (61%)	<.001
Dementia	344 (7%)	40 (2%)	130 (7.7%)	115 (14%)	59 (19%)	<.001
Charlson comorbidity index >2	743 (16%)	65 (3%)	254 (15%)	282 (35%)	142 (46%)	<.001
Haematology and clinical chemistry						
Haemoglobin, g/L	132 [118–145]	139 [127–150]	129 [116–143]	124 [110–136]	119 [106–131]	<.001
Estimated glomerular filtration rate, mL/min¶	70 [49–84]	77 [65–89]	68 [47–82]	53 [36–72]	48 [32–70]	<.001
Peak high sensitivity troponin I, ng/L (log)						
*NSTEMI*	7.02 [5.24–8.63]	7.10 [5.27–8.66]	7.06 [5.32–8.67]	6.92 [5.23–8.61]	6.35 [4.66–8.18]	.02
*STEMI*	9.86 [8.22–10.82]	9.96 [8.35–10.82]	9.85 [8.18–10.82]	9.45 [7.94–10.82]	9.71 [7.98–10.82]	.08
Clinical diagnosis						
NSTEMI	3262 (70%)	1075 (58%)	1264 (74%)	656 (82%)	267 (87%)	<.001
STEMI	1408 (30%)	790 (42%)	435 (26%)	142 (18%)	41 (13%)	
Primary treating speciality						
Cardiology	3146 (67%)	1586 (85%)	1097 (65%)	364 (46%)	99 (32%)	<.001
Medical therapy*						
Aspirin	2587 (65%)	1292 (76%)	837 (59%)	340 (54%)	118 (52%)	<.001
P2Y12 inhibitor	3020 (75%)	1411 (83%)	1044 (73%)	420 (66%)	145 (63%)	<.001
Dual antiplatelet therapy†	2318 (58%)	1208 (71%)	736 (52%)	281 (44%)	93 (41%)	<.001
Anticoagulation‡	340 (9%)	90 (5.3%)	136 (9.5%)	83 (13%)	31 (14%)	<.001
ACE inhibitor or ARB	2067 (52%)	1061 (63%)	695 (49%)	248 (39%)	63 (28%)	<.001
Beta-blocker	2065 (52%)	1013 (60%)	677 (47%)	294 (47%)	81 (35%)	<.001
Lipid lowering therapy	2544 (64%)	1305 (77%)	837 (59%)	303 (48%)	99 (43%)	<.001
Revascularisation						
Coronary angiography	2654 (57%)	1452 (78%)	881 (52%)	265 (33%)	56 (18%)	<.001
Percutaneous coronary intervention	1993 (43%)	1138 (61%)	622 (37%)	195 (24%)	38 (12%)	<.001
Coronary artery bypass grafting	151 (3%)	86 (5%)	46 (3%)	15 (2%)	<5	<.001

Presented as number (%), mean (±SD) or median [25th percentile, 75th percentile].

Abbreviations: SIMD = Scottish Index of Multiple Deprivation, CCI = Charlson comorbidity index, ACE = Angiotensin-converting enzyme; ARB = Angiotensin receptor blocker; NSTEMI = non-ST elevation myocardial infarction; STEMI = ST-elevation myocardial infarction.

^¶^Value missing in 1469 patients.

^*^Restricted to those alive at 30-days post-discharge (overall = 4074; fit = 1707; mild = 1465; moderate = 660; severe = 242).

^†^Two medications from aspirin, clopidogrel, prasugrel and ticagrelor.

^‡^Includes warfarin or novel anticoagulants.

### Patient management

There was an inverse dose–response relationship with frailty severity, with treatment rates lowest in those with severe frailty. Patients with any degree of frailty were less likely to be managed on a cardiology ward, undergo invasive coronary angiography or revascularization, or be prescribed guideline recommended pharmacological therapy (Table 1). Rates of coronary angiography were lowest in those with severe frailty (12% versus 78% fit).

### Primary outcome

**Figure 1:**
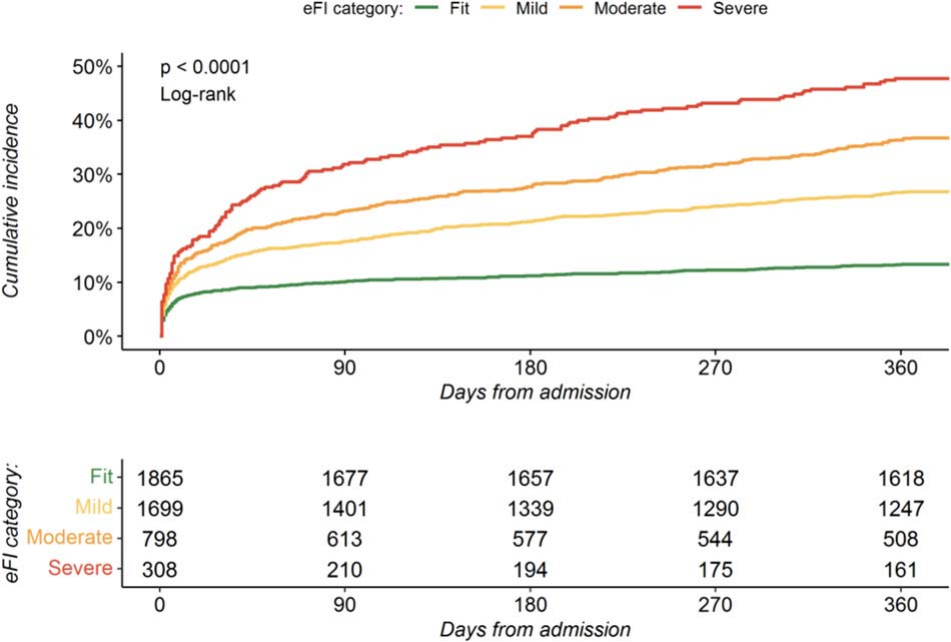
Cumulative incidence of all-cause mortality at 12 months. Cumulative incidence plot of 12-month all-cause mortality by electronic frailty index classification.

**Figure 2:**
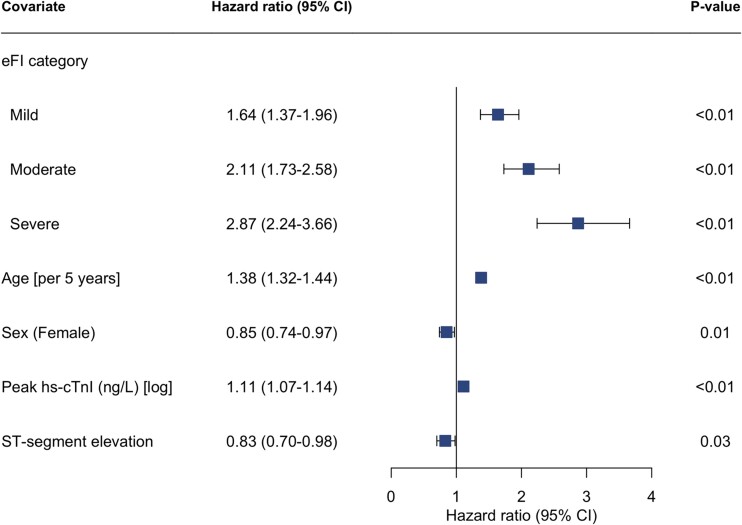
Electronic frailty index and risk of all-cause mortality. Forest plot of cox-regression analysis assessing the associated hazard for all-cause mortality within 12 months of admission by electronic frailty index category (reference = fit). Model adjusted for age, sex, peak troponin value, and myocardial infarction classification (NSTEMI vs STEMI). eFI = electronic frailty index, hs-cTnI = high-sensitivity cardiac troponin I.

In total, 1142 (24%) patients died within 12 months of admission of whom 248 (13%), 454 (27%), 293 (37%) and 147 (47%) were classified as fit, mild, moderate and severely frail respectively (Table 2, Fig. 1). After adjustment, the eFI was independently associated with all-cause death at 12 months, with the risk greatest in those with severe frailty (adjusted hazard ratio [HR] 2.87; 95% confidence interval [CI] 2.24–3.66) (Fig. 2, Supplementary Table S1). The eFI score was adequately calibrated and achieved modest discrimination (AUC 0.67, 95% CI 67–70) for the primary outcome (Supplementary Fig. S1).

**Table 2:** Patient outcomes by electronic frailty index category

	Overall	electronic Frailty Index Category				
		Fit	Mild	Moderate		*P*-value
*N*	4670	1865	1699	798	308	
Primary outcome						
All-cause death at 12 months	1142 (24%)	248 (13%)	454 (27%)	293 (37%)	147 (48%)	<.001
Secondary outcomes						
Duration of stay >5 days	1692 (36%)	531 (28%)	650 (38%)	365 (46%)	146 (47%)	<.001
All-cause death during index presentation	585 (13%)	148 (7.9%)	230 (14%)	137 (17%)	70 (23%)	<.001
Recurrent hospital admission^†^	1253 (27%)	400 (21%)	476 (28%)	275 (34%)	102 (33%)	<.001
*Non-fatal myocardial infarction orurgent revascularisation*	1112 (24%)	379 (20%)	425 (25%)	225 (28%)	83 (27%)	<.001
*Heart failure*	100 (2%)	8 (<1%)	40 (2%)	39 (5%)	13 (4%)	<.001
*Stroke*	18 (<1%)	5 (<1%)	5 (<1%)	5 (<1%)	<5 (<5%)	<.001
*Major bleeding*	67 (1%)	20 (1%)	19 (1%)	21 (3%)	7 (2%)	<.001
Cardiovascular death at 12 months	796 (17%)	190 (10%)	314 (18%)	190 (24%)	102 (33%)	<.001
All-cause death at 3 years*	1698 (36%)	360 (19%)	685 (40%)	446 (56%)	207 (67%)	<.001

Presented as number (%)

Abbreviations: ACE = Angiotensin-converting enzyme; ARB = Angiotensin receptor blocker

^†^due to non-fatal myocardial infarction or urgent revascularisation, heart failure episode, stroke, or major bleeding event within 12 months

^*^Number of patients with minimum 3-year follow-up = 4006 *(eFI category: fit = 1609; mild = 1456; moderate = 676; severe = 265)*

### Secondary outcomes

Frailty was associated with an increased incidence of in-patient death, unplanned hospital admission, cardiovascular mortality at 12 months, and all-cause mortality at 3 years (Table 2). The incidence of each outcome increased in line with escalating frailty category and was highest in those with severe frailty.

**Figure 3:**
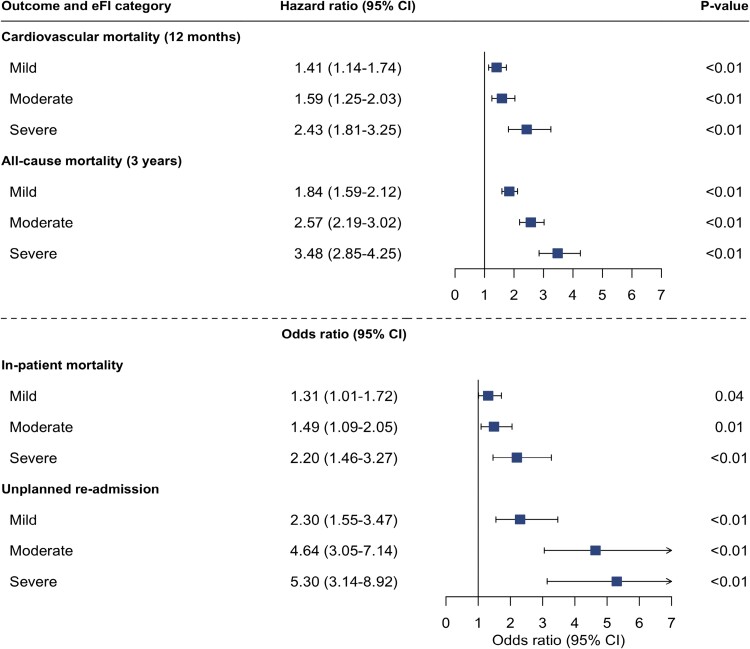
Electronic frailty index and additional adverse outcomes following myocardial infarction. Forest plot showing output of cox-regression and logistic regression analysis assessing the association of electronic frailty index categories with cardiovascular mortality within 12 months, all-cause mortality at 3 years, in-patient all-cause mortality, and unplanned hospital re-admission within 12-months of discharge. Adjusted for age, sex, peak troponin value and myocardial infarction classification (NSTEMI vs STEMI).

Any degree of frailty was associated with an increased odds of both in-patient death and unplanned hospital admission with the odds greatest in those with severe frailty (reference = fit; adjusted odds ratio [OR] 2.20 [95% CI 1.46–3.27] and OR 5.30 [95% CI 3.14–8.92], respectively, *P* < .001 for both) (Fig. 3, Supplementary Table S2).

Cardiovascular death was the primary cause of death across all frailty categories. In total, 796 (70%) of all deaths were due to a cardiovascular cause of which 190 (10%) and 102 (33%) occurred in fit and severely frail patients, respectively. Patients with severe frailty had a persisting but attenuated risk of cardiovascular death (adjusted HR 2.43 [95% CI 1.81–3.25]) (Fig. 3, Supplementary Table S3).

At 3 years, 207 (67%) patients classified as severely frail had died compared with 360 (19%) classified as fit. The risk of death within 3 years was greatest in those with severe frailty (adjusted HR 3.48; 95% CI 2.85–4.25) (Fig. 3, Supplementary Table S3).

### Subgroup analysis

The associated hazard of all-cause death was similar across frailty categories when stratified by sex or a final diagnosis of non-ST-segment elevation or ST-segment elevation myocardial infarction. In patients classified as severely frail, those aged <75 years had a two-fold adjusted increase in the risk of all-cause mortality at 12 months compared to those aged ≥75 years (HR 5.17 [95% CI 2.94–9.12] versus 2.48 [95% CI 1.88–3.26]) (Supplementary Fig. S2).

## Discussion

In 4670 consecutive older patients with a diagnosis of myocardial infarction, we have measured frailty using the eFI and determined its relationship with key outcomes. We report several findings important to clinical practice. First, frailty is common in older patients with myocardial infarction, affecting over half of our study population of which 1 in 10 were classified as severely frail. Second, we were able to quantify frailty in all enrolled patients using linked routine electronic healthcare data. Third, frailty classified using the eFI was independently associated with key adverse outcomes following myocardial infarction including short and long-term all-cause mortality. Over half of patients classified as severely frail died within 12 months, increasing to more than three quarters at three years. Our findings highlight the scale and impact of frailty in older patients with myocardial infarction and the potential value of using routinely available healthcare data to systematically calculate prognostic tools in real-time to inform clinical care.

Our understanding of cardiovascular disease has evolved rapidly over the last decade resulting in a reduction in age-specific cardiovascular mortality [23, 24]. In comparison, our understanding of how best to manage frail patients has changed little. Research to date has focused on the treatment of single disease processes in isolation despite the majority of patients encountered in clinical practice suffering from multiple interacting health conditions. Population ageing and the resulting increase in multimorbidity and frailty will profoundly impact healthcare services. If we are to develop tools to aid the management of these complex patients, we first need a valid, efficient and easily implementable measure of frailty that corresponds to meaningful patient outcomes.

We were able to calculate the eFI in all patients and demonstrate an independent association with the risk of both all-cause mortality and cardiovascular specific outcomes. Our finding of an approximately 3-fold adjusted increase in the risk of death within 1-year in those classified as severely frailty is consistent with evaluations using other frailty assessment tools [4, 12, 25–27]. Importantly, an increased risk of death was observed across all frailty categories, including in those classified with mild frailty, and was irrespective of age, sex, or markers of infarct severity. Interestingly, we observed that a classification of severe frailty conferred a two-fold greater risk of all-cause death in patients under 75 years compared to those aged 75 years and over, a finding reported by others and one that highlights the utility of frailty assessment outside the very old [4].

In our cohort of consecutive patients, over half were classified as frail using the eFI. This proportion is likely to increase over the coming decades. The prevalence of frailty in patients with cardiovascular disease varies considerably, from 4.7% in clinical trial populations to 60% in observational registries [28, 29]. The eFI includes several risk factors for coronary artery disease, which may lead to a greater proportion of patients within our cohort being classified as frail. However, this reflects the importance of cardiovascular disease as both a cause and consequence of frailty [30, 31].

As observed previously, patients with frailty were less likely to receive invasive management in comparison to non-frail patients with fewer than 1 in 10 severely frail patients undergoing angiography compared to in 8 in 10 non-frail patients [4, 32, 33]. There are several explanations for this. First, older frail patients are less likely to present with typical symptoms or signs of ischaemia on 12-lead electrocardiogram and the specificity of high-sensitivity cardiac troponin is decreased making the diagnosis challenging [34]. Second, older adults are under-represented in randomised trials and the benefits and risks of established therapies, as well as how these factors are attenuated by co-existing multimorbidity and frailty, are unclear [35–40]. Finally, there may be a perception that any benefit gained from therapies is outweighed by the competing risk of death or disability as a result of non-cardiovascular conditions more common in an older population that are not modifiable by available cardiovascular therapies. Our observation that the majority of deaths across all frailty categories were due to cardiovascular causes, with a comparatively low rate of non-cardiovascular death that remained constant following admission, challenges this concept and highlights the previously described treatment paradox: those who are at greatest risk of cardiovascular death are the least likely to receive treatment proven to reduce this outcome. Further trials including patients more representative of those seen in clinical practice are needed to ascertain optimal treatment strategies for frail older adults.

There is currently no gold standard method of frailty assessment [41]. We chose to assess the eFI for several reasons. First, frailty assessment using electronic health care data has several advantages over traditional in person assessment, notably reduced risk of observer bias, ease of implementation, reproducibility, and the potential to measure frailty at scale with minimal resources by embedding automatic calculation within digital health records. This approach is feasible, with automatic calculation of the eFI within digital health records already successfully implemented across the National Healthcare Service in the United Kingdom. Second, the eFI uses primary care Read codes. Frailty is a result of impairment across multiple domains including deficits in physical status which may not be captured when using secondary care ICD-10 codes alone. Third, the greater frequency of interaction with primary care services enables repeat calculation over time, allowing changes in frailty status to be observed, which could offer insight on the impact of specific interventions, such as cardiac rehabilitation. Finally, the eFI has been successfully translated and used in international cohorts with alternative primary care coding systems, increasing the utility and applicability of our findings [42].

It remains unclear how the growing body of evidence demonstrating the importance of frailty in patients with myocardial infarction should be translated in to meaningful improvements in clinical care. Although the eFI provided important prognostic information, we observed only modest discrimination for all-cause mortality, suggesting its role in individualised risk stratification may be limited [43]. Discrimination was lower than that achieved in contemporary cohorts when applying the current gold standard cardiovascular risk prediction tool, the GRACE score [8, 44, 45]. This is not surprising, given the eFI was not designed as an individual risk prediction tool. However, the addition of frailty measures to the GRACE score has been shown to improve risk prediction in older patients. [46–48] The ability to routinely identify the most vulnerable patients, at the time of admission, could aid the use of targeted frailty intervention services, inform treatment goals, or prompt discussions on advanced care planning to optimise quality and end-of life care [49]. However, such approaches would depend upon integrated primary and secondary electronic health record systems that are difficult to implement. Tailored cardiac rehabilitation programmes have demonstrated modest improvement in frailty measures in patients with cardiovascular disease [9]. Whether interventions to reduce or impede the progression of frailty can prevent the escalating risk of death seen in those with more pronounced symptoms remains unclear.

### Study limitations

There are several limitations of this study. First, our analysis is restricted to patients treated in Scotland and may not be representative of other healthcare systems. However, our findings are in keeping with those from comparable studies across a variety of geographical settings [4, 32, 47]. Second, while the accuracy of ICD-10 coding is regularly audited and has been shown to be of a standard sufficient for use in research, we cannot exclude cases of misclassification [50]. Third, data were limited to variables present within the DataLoch Heart Disease Registry. We were therefore unable to assess the impact of additional key variables known to be associated with prognosis, such as ejection fraction and GRACE score. Fourth, the eFI relies on patients interacting with their primary care physician and the accurate documentation of conditions. This may result in under reporting of frailty and misclassification of severity. Lastly, we were unable to explore the relationship between the eFI and other outcomes of interest, such as functional or cognitive decline.

## Conclusions

In conclusion, the electronic Frailty Index identified patients at increased risk of death and major adverse cardiovascular events following myocardial infarction. Measurement of frailty from linked healthcare data is feasible and could provide a low-cost method of identifying vulnerable older patients on hospital presentation.

## Supplementary Material

aa-24-0117-File002_afae150
