# Supplementary material for: The electronic frailty index and outcomes in patients with myocardial infarction
Source: Age Ageing. 2024 Jul 16;53(7):afae150. doi: 10.1093/ageing/afae150 (PMC11249914; doi:10.1093/ageing/afae150)

APPENDIX MATERIALS

**The electronic frailty index and outcomes in patients**

**with myocardial infarction**

**Appendix methods**

**Domains used to calculate the electronic frailty index**

| **DOMAINS** | | | |
| --- | --- | --- | --- |
| **Disease States** | **Symptoms & signs** | **Disability** | **Laboratory** |
| Arthritis | Dizziness | Activity limitation | Anaemia/haematinic deficiency |
| Atrial fibrillation | Dyspnoea | Hearing impairment |  |
| Cerebrovascular disease | Falls | Housebound |  |
| Chronic kidney disease | Foot problems | Mobility and transfer problems |  |
| Diabetes | Fragility fracture | Polypharmacy |  |
| Heart failure | Memory and cognitive problems | Requirement for care |  |
| Heart valve disease | Sleep disturbance | Social vulnerability |  |
| Hypertension | Urinary incontinence | Visual impairment |  |
| Hypotension/syncope | Weight loss and anorexia |  |  |
| Ischaemic heart disease |  |  |  |
| Osteoporosis |  |  |  |
| Parkinsonism and tremor |  |  |  |
| Peptic ulcer |  |  |  |
| Peripheral vascular disease |  |  |  |
| Respiratory disease |  |  |  |
| Skin ulcer |  |  |  |
| Thyroid disease |  |  |  |
| Urinary system disease |  |  |  |

**Description of data sources**

Baseline comorbidities were identified using primary and secondary care data read codes. The presence or previous diagnosis of a condition was defined using CALIBER Portal algorithms and code lists with codes eligible for inclusion if they were present 24 hours prior to the index admission.[19] National prescribing records were used to identify pre-admission medications and medications present on community prescriptions at 30 days post discharge. Haematology and clinical biochemistry results were available through linkage to local and regional laboratory services. ICD-10 and Office of Population Censuses and Surveys Classification of Interventions and Procedures (OPCS-4) codes were used to identify in-patient procedures and operations.

1. Kuan V, Denaxas S, Gonzalez-Izquierdo A, Direk K, Bhatti O, Husain S, et al. A chronological map of 308 physical and mental health conditions from 4 million individuals in the English National Health Service. The Lancet Digital Health 2019;1:e63-e77. doi: 10.1016/S2589-7500(19)30012-3

**Data availability statement**

The study data, analysis code and DataLoch registry can be accessed by individuals who have undertaken the necessary governance training on application to DataLoch (<https://dataloch.org>). Summary data can be made available upon request to the corresponding author.

**Appendix Tables**

1. **Supplementary Table 1: All-cause mortality at 12 months**
2. **Supplementary Table 2: In-patient mortality and unplanned re-admission within 12 months**
3. **Supplementary Table 3: Cardiovascular mortality at 12 months and all-cause mortality 3 years**

**Supplementary Table 1: All-cause mortality at 12 months**

Output of univariable and multivariable cox regression for the outcome of all-cause mortality within 12 months of admission

| Co-variates | Univariable model  HR (95% CI) | Multivariable model  HR (95% CI) |
| --- | --- | --- |
| Electronic frailty index |  |  |
| Fit | - | - |
| Mild | 2.15 (1.84-2.51, p<0.001) | 1.64 (1.37-1.96, p<0.001) |
| Moderate | 3.11 (2.62-3.68, p<0.001) | 2.11 (1.73-2.58, p<0.001) |
| Severe | 4.33 (3.53-5.31, p<0.001) | 2.87 (2.24-3.66, p<0.001) |
| Age [per 5 years] | 1.45 (1.40-1.50, p<0.001) | 1.38 (1.32-1.44, p<0.001) |
| Sex (Female) | 1.16 (1.03-1.30, p=0.012) | 0.85 (0.74-0.97, p=0.013) |
| Peak hs-cTnI (ng/L) [log] | 1.05 (1.02-1.08, p<0.001) | 1.11 (1.07-1.14, p<0.001) |
| ST-segment elevation | 0.70 (0.61-0.80, p<0.001) | 0.83 (0.70-0.98, p=0.025) |
| Output displayed as hazard ratio (HR) (95% confidence interval (CI))  Hs-cTnI = High-sensitivity cardiac troponin I | | |

**Supplementary Table 2: In-patient mortality an unplanned re-admission within 12 months**

Output of univariable and multivariable logistic regression for an outcome of in-patient all-cause mortality and unplanned re-admission within 12 months

| Outcome | In-patient mortality | | Unplanned re-admission within 12 months | |
| --- | --- | --- | --- | --- |
| Co-variates | **Univariable model**  **OR (95% CI)** | **Multivariable model**  **OR (95% CI)** | **Univariable model**  **OR (95% CI)** | Multivariable model  OR (95% CI) |
| Electronic frailty index |  |  |  |  |
| Fit | - | - | - | - |
| Mild | 1.82 (1.46-2.26, p<0.001) | 1.31 (1.01-1.72, p=0.044) | 2.37 (1.65-3.46, p<0.001) | 2.30 (1.55-3.47, p<0.001) |
| Moderate | 2.40 (1.87-3.08, p<0.001) | 1.49 (1.09-2.05, p=0.01) | 4.98 (3.44-7.33, p<0.001) | 4.64 (3.05-7.14, p<0.001) |
| Severe | 3.41 (2.48-4.66, p<0.001) | 2.20 (1.46-3.27, p<0.001) | 5.43 (3.40-8.63, p<0.001)) | 5.30 (3.14-8.92, p<0.001) |
| Age [per 5 years] | 1.43 (1.35-1.51, p<0.001) | 1.38 (1.29-1.48, p<0.001) | 1.29 (1.19-1.39, p<0.001) | 1.15 (1.06-1.26, p=0.001) |
| Sex (Female) | 1.24 (1.05-1.48, p=0.014) | 0.87 (0.70-1.07, p=0.192) | 1.06 (0.82-1.37, p=0.664) | 0.81 (0.61-1.07, p=0.139) |
| Peak hs-cTnI (ng/L) [log] | 1.09 (1.04-1.14, p<0.001) | 1.12 (1.06-1.19, p<0.001) | 1.04 (0.98-1.10, p=0.200) | 1.11 (1.04-1.19, p=0.002) |
| ST-segment elevation | 0.82 (0.68-1.00, p=0.050) | 1.05 (0.82-1.36, p=0.682) | 0.62 (0.45-0.83, p=0.002) | 0.71 (0.49-1.00, p=0.054) |
| Output displayed as odds ratio (OR) (95% confidence interval (CI))  Hs-cTnI = High-sensitivity cardiac troponin I | | | | |

**Supplementary Table 3: Cardiovascular mortality at 12 months and All-cause mortality 3 years**

Output of univariable and multivariable competing risk cox regression for the outcome of cardiovascular mortality within 12 months of admission and univariable and multivariable cox regression for the outcome of all-cause mortality within 3 years of admission

| Outcome | Cardiovascular mortality at 12 months | | All-cause mortality 3 years | |
| --- | --- | --- | --- | --- |
| Co-variates | **Univariable model**  **HR (95% CI)** | **Multivariable model**  **HR (95% CI)** | **Univariable model**  **HR (95% CI)** | Multivariable model  HR (95% CI) |
| Electronic frailty index |  |  |  |  |
| *Fit* | - | - | - | - |
| *Mild* | 1.88 (1.57-2.25, p<0.001) | 1.41 (1.14-1.74, p<0.001) | 2.38 (2.10-2.71, p<0.001 | 1.84 (1.59-2.12, p<0.001) |
| *Moderate* | 2.48 (2.03-3.03, p<0.001) | 1.59 (1.25-2.03, p<0.001) | 3.67 (3.19-4.22, p<0.001 | 2.57 (2.19-3.02, p<0.001) |
| *Severe* | 3.61 (2.85-4.57, p<0.001) | 2.43 (1.81-3.25, p<0.001) | 4.96 (4.18-5.88, p<0.001 | 3.48 (2.85-4.25, p<0.001) |
| Age [per 5 years] | 1.08 (1.07-1.08, p<0.001) | 1.39 (1.32-1.46, p<0.001) | 1.46 (1.42-1.51, p<0.001) | 1.37 (1.33-1.42, p<0.001) |
| Sex (Female) | 1.14 (0.99-1.31, p=0.069) | 0.85 (0.72-1.00, p=0.043) | 1.15 (1.05-1.27, p=0.004 | 0.82 (0.74-0.91, p<0.001) |
| Peak hs-cTnI (ng/L) [log] | 1.07 (1.03-1.11, p<0.001) | 1.11 (1.07-1.16, p<0.001) | 1.03 (1.01-1.05, p=0.008) | 1.09 (1.06-1.12, p<0.001) |
| ST-segment elevation | 0.78 (0.67-0.92, p<0.003) | 0.89 (0.72-1.10, p=0.300) | 0.69 (0.62-0.77, p<0.001 | 0.84 (0.74-0.96, p=0.012) |
| Output displayed as hazard ratio (HR) (95% confidence interval (CI))  Hs-cTnI = High-sensitivity cardiac troponin I | | | | |

**Appendix Figures**

1. **Supplementary Figure 1: Discrimination and calibration**
2. **Supplementary Figure 2: Subgroup analysis**

**Supplementary Figure 1: Discrimination and calibration**

Discrimination (A) and calibration (B) of the electronic frailty index score for an outcome of 12-month all-cause mortality


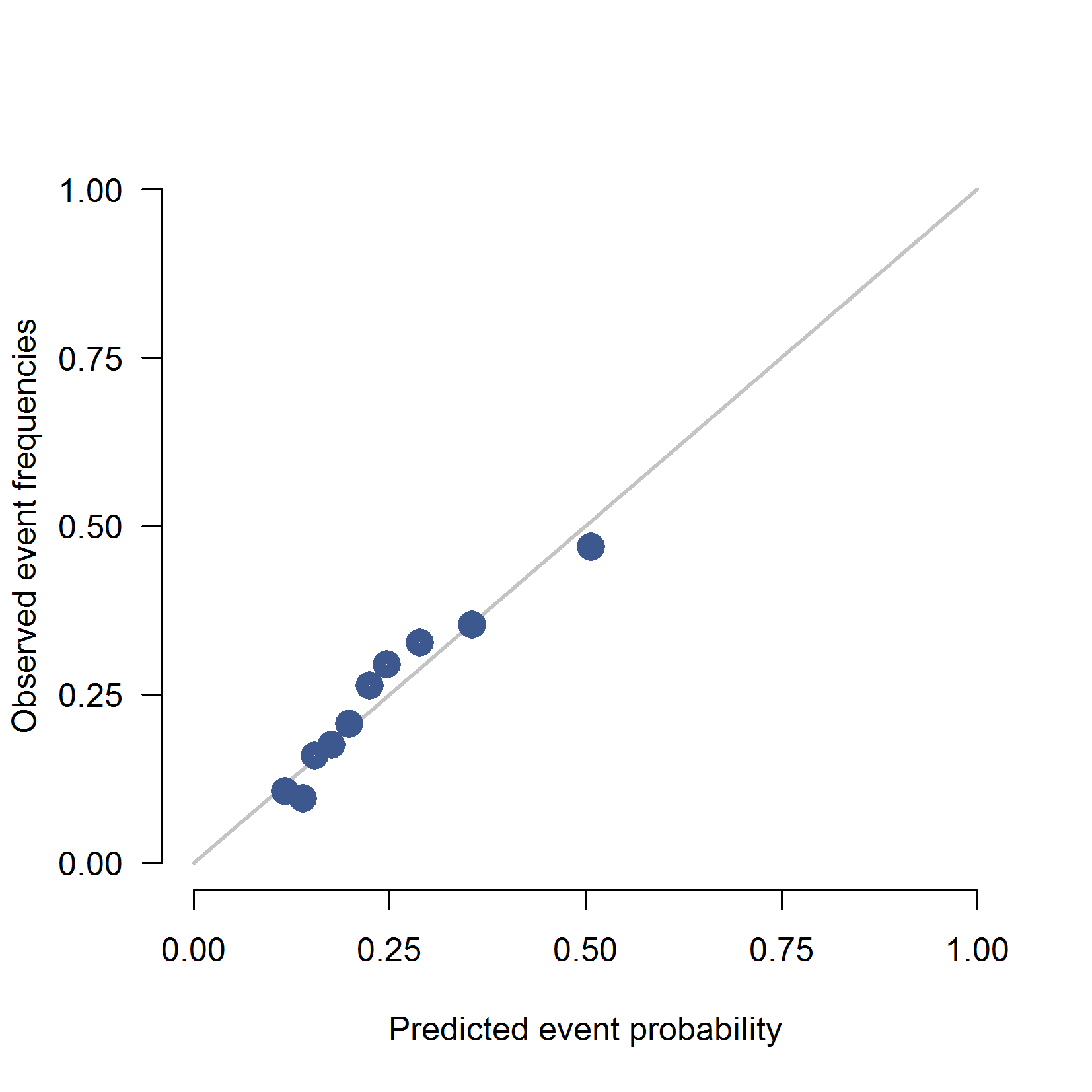

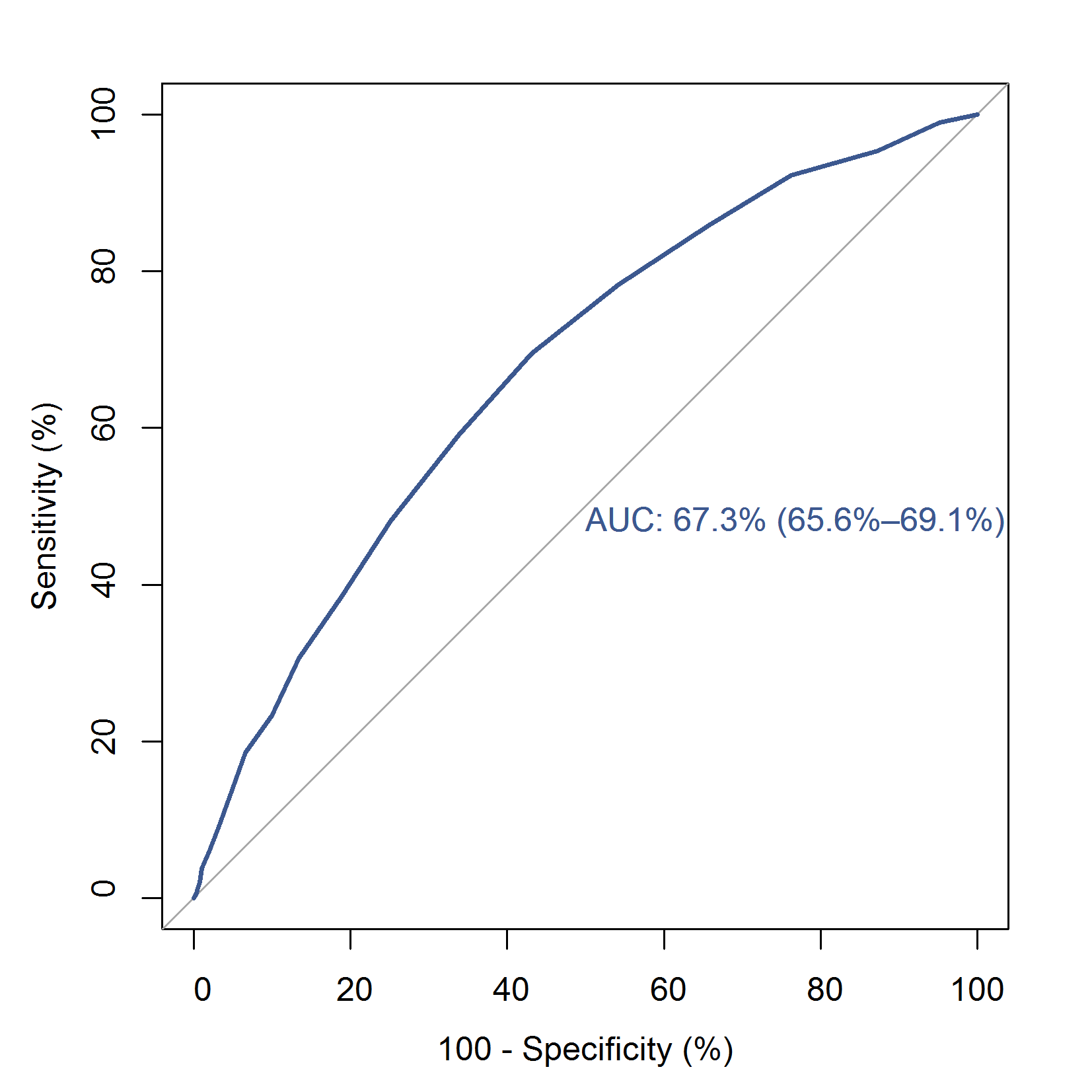


**A**

**B**

**Supplementary Figure 2: Subgroup analysis**

Cox-regression analysis for the primary outcome of all-cause mortality at 12 months by key patient subgroup. Models adjusted for age, sex, maximal cardiac troponin value and myocardial infarction classification (STEMI *versus* NSTEMI).
*P-value for interaction


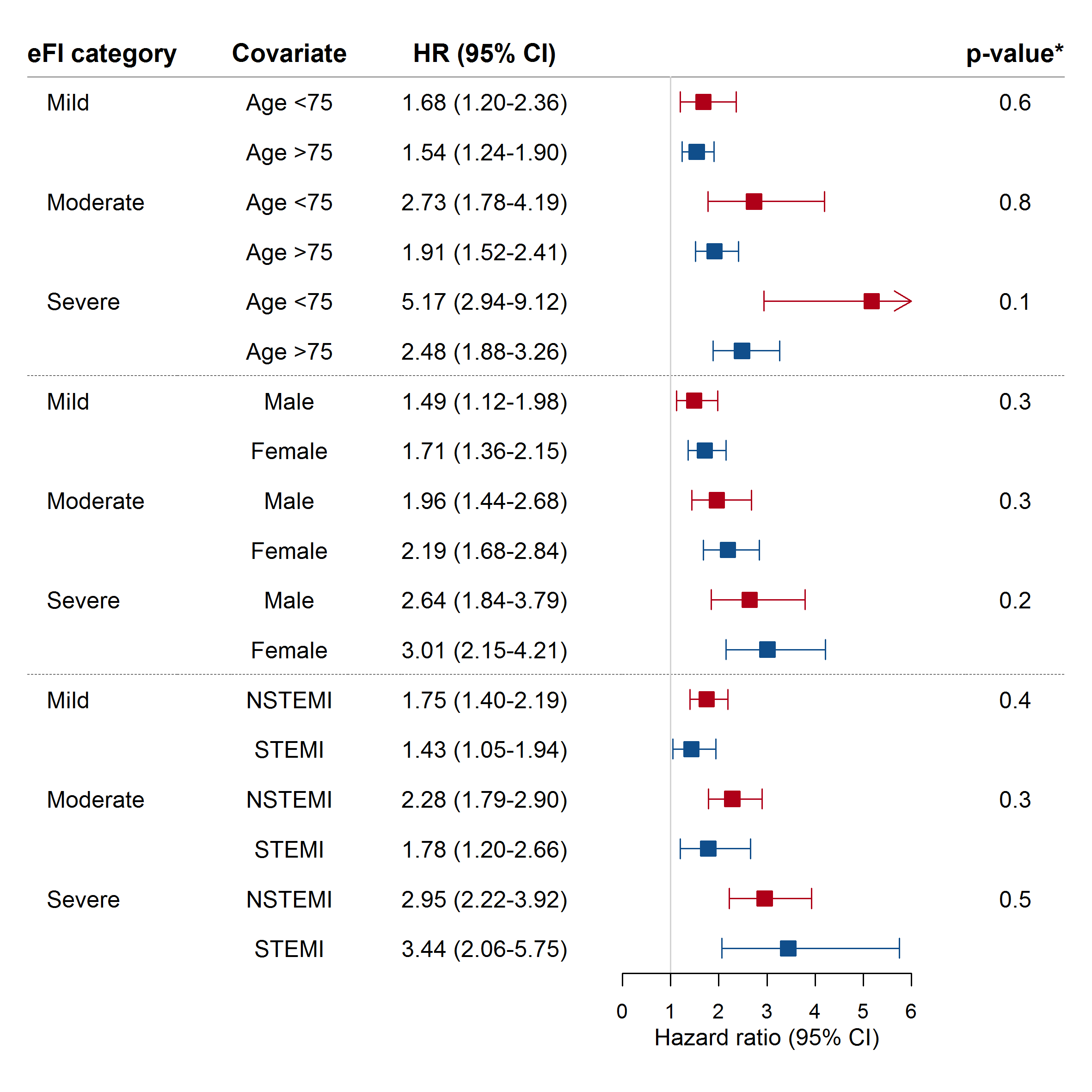

Supplement: aa-24-0117-File002_afae150 [file aa-24-0117-file002_afae150.docx]
